# Impact of inoculum density of *Fusarium oxysporum* f. sp. *zingiberi* on symptomatic appearances and yield of ginger (*Zingiber officinale* Roscoe)

**DOI:** 10.1099/acmi.0.000605.v3

**Published:** 2023-09-11

**Authors:** Andrea Matthews, Sharan P. T. Muthukumar, Sharon Hamill, Elizabeth A. B. Aitken, Andrew Chen

**Affiliations:** ^1^​ School of Agriculture and Food Sustainability, The University of Queensland, St Lucia, QLD 4067, Australia; ^2^​ Department of Agriculture and Fisheries, Maroochy Research Facility, Nambour, QLD 4560, Australia

**Keywords:** crop yield, *Fusarium oxysporum* f. sp. *zingiberi*, Fusarium yellows, ginger, inoculum dose, *Zingiber officinale *Roscoe

## Abstract

Ginger (*Zingiber officinale* Roscoe) is an important horticultural crop valued for its medicinal and culinary properties. Fusarium yellows, caused by the ascomycete fungus *Fusarium oxysporum* f. sp. *zingiberi* (*Foz*), is a devastating soil-borne disease of ginger. It has curtailed ginger production in Australia and around the world, leading to significant economic losses. An integrated approach is required to manage soil-borne diseases such as those caused by *Foz*. However, little is known about the influence of *Foz* inoculum on disease severity. This study aimed to establish a minimum threshold level of spores per gram of soil required for plant infection and to develop and evaluate a pot inoculation method for screening large numbers of plants in a controlled environment. To achieve this, the dominant Australian ginger cultivar Canton was inoculated with 10^1^, 10^3^, 10^5^, 10^6^ and 10^7^ microconidia g^−1^ soil. The inoculum density was positively associated with leaf and stem yellows, and rhizome discolouration, and negatively associated with root length and rhizome weight. The lowest threshold required for plant infection was 10^1^ microconidia g^−1^ soil, which may provide an important basis for outbreaks of *Foz* in the field. This finding adds significantly to our knowledge of the impact of soil health on ginger production, thereby contributing to the integrated management of *Foz*. When used at a high dose, this method can facilitate reliable and accurate screening of *Foz*-susceptible ginger genotypes in a controlled environment.

## Data Summary

The authors confirm all supporting data, code and protocols have been provided within the article.

**Impact Statement**

Phenotypic screening of plants is required for the identification of host resistance against agronomically important soil-borne pathogens. In the case of *Foz*, a reliable phenotyping method needs to account for environmental variations as well as the sporadic expression of *Foz* symptoms. By establishing a consistent methodology for optimal parameters for plant growth, we intended to determine the level of inoculum dose required to induce uniform *Foz* infection. The outcomes of this study will aid future research into ginger infection by *Foz* and help us to understand the threshold level of *Foz* that is required to cause an outbreak in the field, potentially contributing to an integrated approach to manage this important disease.

## Introduction

Ginger (*Zingiber officinale* Roscoe) is an important horticultural crop valued for its medicinal and culinary properties. It has traditionally been used to reduce nausea, provide remedies for influenza and the common cold, and aid digestion [1]. In food preparations, ginger has been widely used as a cooking spice, and it is also consumed as ginger ale and tea. Although ginger is consumed all over the world, its cultivation is limited to the tropics and subtropics, with a global production of over 200 000 tonnes of ginger rhizomes per annum [2]. In Australia, the ginger industry has a market value of AU $116 million per annum [3], with production primarily based in southeast Queensland.

Globally, ginger production is threatened primarily by rhizome rot or root rot diseases caused by seed and soil-borne pathogens such as *Fusarium* spp. and *Pythium* spp., with these being major contributing factors in yield reduction [4–6]. *Fusarium oxysporum* strains infect hundreds of plant species, producing symptoms such as wilts and yellows. However, each strain, or *formae specialis* (f. sp.), is host-specific and is generally named based on the host to which it is pathogenic [7]. *Fusarium oxysporum* f. sp. *zingiberi* (*Foz*) is pathogenic against ginger, and is known by the common names Fusarium yellows [4] or Fusarium wilt [8]. External symptoms of Fusarium yellows include stunted growth and yellowing and drooping of older leaves, leading to senescence of individual tillers [9]. However, it is the internal discolouration and rotting of the rhizomes that is associated with the reduction of ginger rhizome size and quality. Internally, this brown discolouration is observed in the water-conducting regions of the rhizome and rotting of the tissues in the rhizome cortex [9].

Once the soil is infected, *Foz* is thought to survive as chlamydopores in the absence of a susceptible ginger host and hence can persist for years in the form of these resting spores in the soil [10]. The inoculum concentrations in this study only refer to microconidia. It has not been established if macroconidia or chlamydospores would produce similar results. Chlamydospores, as long-term survival structures, may be the primary source of infection after an extended absence of ginger and it would be particularly beneficial to better understand the role of these other spores in infection cycles.

Commercial production of ginger in Australia relies predominantly on two commercial ginger cultivars, Canton and Queensland [11]. However, both cultivars are susceptible to *Foz* [9, 12]. The need to address the lack of *Foz-* and other pathogen-resistant genotypes in the Australian ginger industry [13] is an important priority for research and investment [14]. It is therefore important that an efficient method for screening ginger cultivars for resistance to pathogens such as *Foz* is developed to expedite elite cultivar release.

To study the efficacy of *Foz* infection, we performed an experiment using five different concentrations of *Foz* microconidia and analysed the effect of soil spore concentration on disease development in the ginger cultivar Canton, which is the main culinary cultivar produced in Australia [9]. The impact of *Foz* infection on ginger plants was apparent, with symptoms including leaf yellowing and reduced rhizome weight. *Foz* was detected on plants subjected to as little as 10 microconidia g^−1^ soil. Symptom severity was positively associated with the amount of spores in the inoculum and negatively associated with plant development. The outcome from this study contributes to our understanding of the threshold levels of *Foz* required to cause an outbreak of Fusarium yellows in the field as well as provide an optimized methodology for screening ginger genotypes for *Foz* resistance as new cultivars are developed in the future.

## Full-Text

## Methods

Four *Fusarium oxysporum* strains (UQ6848, UQ6851, UQ6852 and UQ6853) were isolated from ginger plants symptomatic for yellows from the following locations in southern Queensland, Australia: Gympie (one), Bundaberg (one) and Nambour (two). *Formae specialis* of these isolates were confirmed to be *Fusarium oxysporum* f. sp. *zingiberi* based on their ability to cause Fusarium yellows in ginger pot trials and by phylogenetic analysis based on the translation elongation factor 1-alpha (*TEF-1α*) gene of the isolates [15]. Monoconidial isolates were subsequently reisolated using hyphal tips of single germinated conidia. Spore suspensions were prepared for the four isolates according to a previously published method [9] and then mixed in equal ratios of spore concentrations prior to plant inoculation. The isolates were mixed in equal proportions to allow for differences in virulence between isolates. To generate the spore suspensions, flasks of potato dextrose broth were inoculated with each of the isolates and were incubated for 7 days at 26 °C with shaking at 120 r.p.m. on a rotating platform (Ratek, Victoria, Australia). The spores were then extracted, counted and adjusted using sterile water to 10^2^, 10^4^, 10^6^, 10^7^ and 10^8^ microconidia ml^−1^ for inoculation on ginger plants, as per a previous study [9].

DNA was extracted from the mycelia of the four isolates using a microwave method [16]. *TEF-1α* was PCR-amplified using published primers (5′-ATGGGTAAGGARGACAAGAC) and (5′-GGARGTACCAGTSATCATGTT) [15] and DreamTaq (Thermo Fisher Scientific, Waltham, MA, USA). A single product of 656 bp was visualized on a 1 % agarose gel and then purified using a GeneJET PCR purification kit (Thermo Fisher Scientific, Waltham, MA, USA), and Sanger-sequenced at the Australian Genome Research Facility, Melbourne, Australia.

*TEF-1α* sequences representing 156 isolates belonging to four *Fusarium* species complexes were retrieved from previous studies [9, 15, 17, 18]. Geneious Prime v 2023.0.1 (Biomatter Pty Ltd, Auckland, New Zealand) was used for the phylogenetic reconstruction of the isolates. Firstly, multiple sequence alignment was performed using MAFFT v 7.490 [19]. The subsequent alignment was used to pick regions with minimized gaps and derive a consensus alignment sequence of 543 bp, which was then used as an input in MrBayes v 3.2.6 to reconstruct the phylogeny using the Bayesian inference method [20]. The running parameters used the GTR-G-I model of substitution with two independent analyses on four Markov chain Monte Carlo (MCMC) chains for 2 000 000 generations. A burn-in rate of 25 % was used to sample every 1000 generations. *Fusarium delphinoides* (NRRL36160) was used as an outgroup to anchor the phylogenetic tree. The tree branches were transformed into a cladogram and visualized in Geneious Prime.

**Fig. 1.**
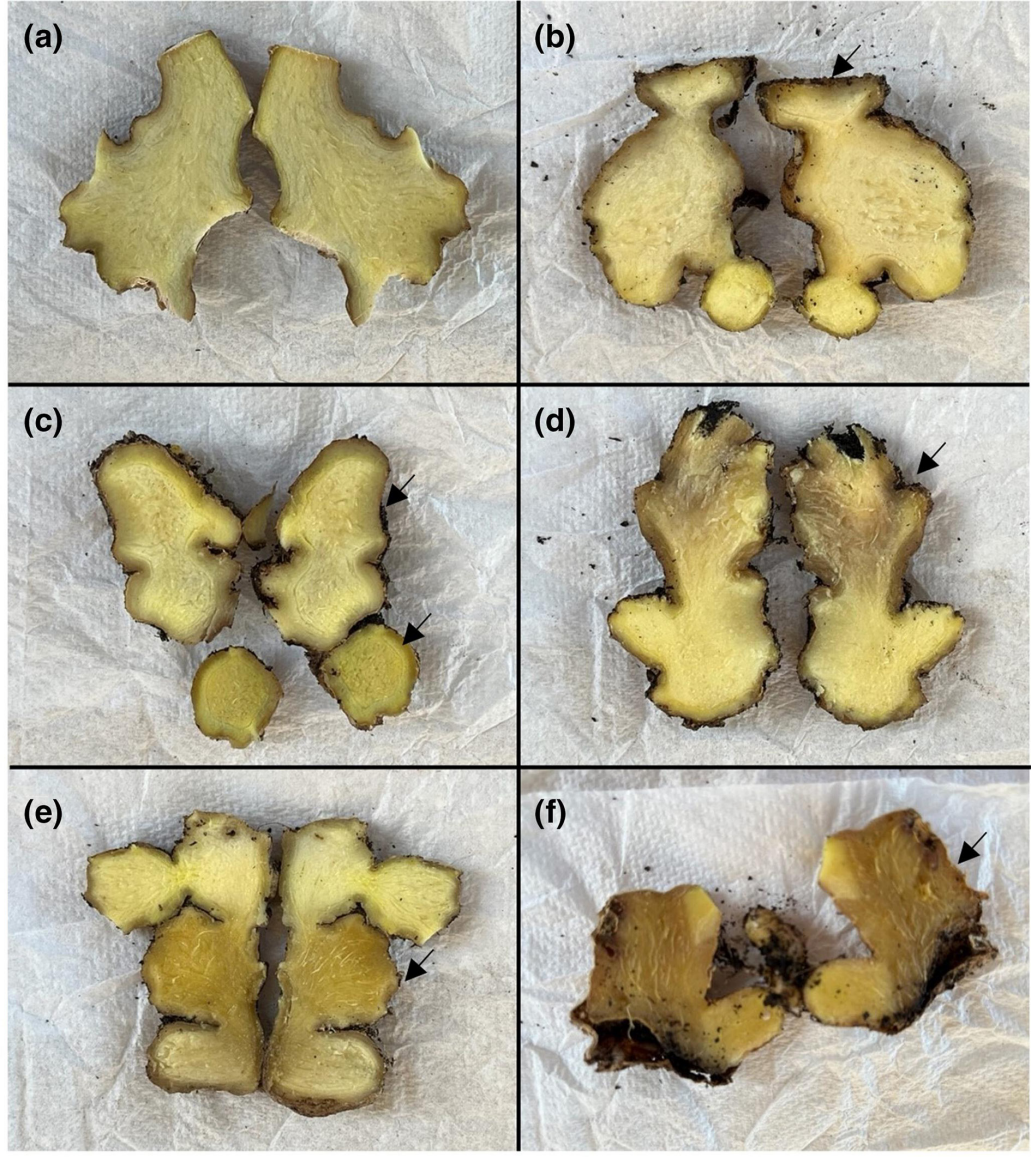
Ginger rhizome discolouration index used to score the internal symptoms of the diseased plants in this study. The scale was (**a**) completely clean, no discolouration observed; (**b**) less than 10 % discolouration including edge effects or small patches, where it is not completely clean; (**c**) brown discoloured areas occupying over 10 % and up to 25 % of the rhizome; (**d**) brown discoloured areas occupying over 25 % and up to 50 % of the rhizome; (**e**) brown discoloured areas occupying over 50 % and up to 75 % of the rhizome; (**f**) brown discoloured areas occupying over 75 % and up to 100 % of the rhizome. Arrows indicate the visible regions of discolouration in the rhizomes.

The ginger plantlets of cultivar Canton were produced by *in vitro* tissue culture [21, 22] and then grown in a glasshouse to maintain a disease-free state at the Maroochy Research Facility, Queensland, Australia. A quantity of approximately 400 g of a premium potting mix (Searles, Kilcoy, Queensland, Australia) was used per 100 mm (L) × 100 mm (W) × 120 mm (D) square pot. In total, 53 plants of the susceptible ginger cultivar Canton at a stem height of 15–20 cm carrying 8–12 leaves each were transferred to a Conviron GEN1000 TA plant growth chamber (Winnipeg, MB, Canada) at the University of Queensland, St Lucia, Australia. The growth parameters include a 14 h light/10 h night photoperiod regime with lights set to 100 % intensity at a photon irradiance rate of 700 µmol m^−²^ s^−1^, and day and night temperatures kept at 27 and 24 °C, respectively. Watering was done three times a week. Plants were climatized in the chamber for 1 week. For each pot, 40 ml of a concentrated spore suspension was mixed with 400 g of soil and the plant was potted into the inoculated soil. The final spore concentrations were 10^1^, 10^3^, 10^5^ and 10^7^ microconidia g^−1^ of soil, respectively. Control plants were mock inoculated with sterile water. All but the 10^7^ microconidia g^−1^ of the soil treatment group had 10 plants each. For the 10^7^ microconidia g^−1^ of the soil treatment group, due to a limited availability of plants, only three plants were used. Each pot was self-contained in a zip-lock bag and grouped with replicates. External and internal symptoms were assessed 8 weeks post-inoculation. Interval symptom was scored using a 1–6 severity scale based on the visual identification of discoloured regions in the rhizome (Fig. 1). Reisolation of *Foz* in the rhizome, roots and tillers was performed according to a previous study [9].

The statistical software SPSS v28.0.1.0 (142) (IBM Corp., Armonk, NY, USA) was used to perform one-way analysis of variance (ANOVA) in a pair-wise manner, with measurements for the various traits set as dependent variables and the treatments as the categorical variable. Waller–Duncan’s multiple range testing was performed as a post-hoc test to separate the means into subsets by least significant difference (LSD). The harmonic mean sample size of 7.2 was estimated and used to account for the unequal variance associated with the uneven sample size (*n*) of the treatments. The type 1/type 2 error seriousness ratio (k-ratio) was set to 100 (α=0.05).

## Results and discussion

**Fig. 2.**
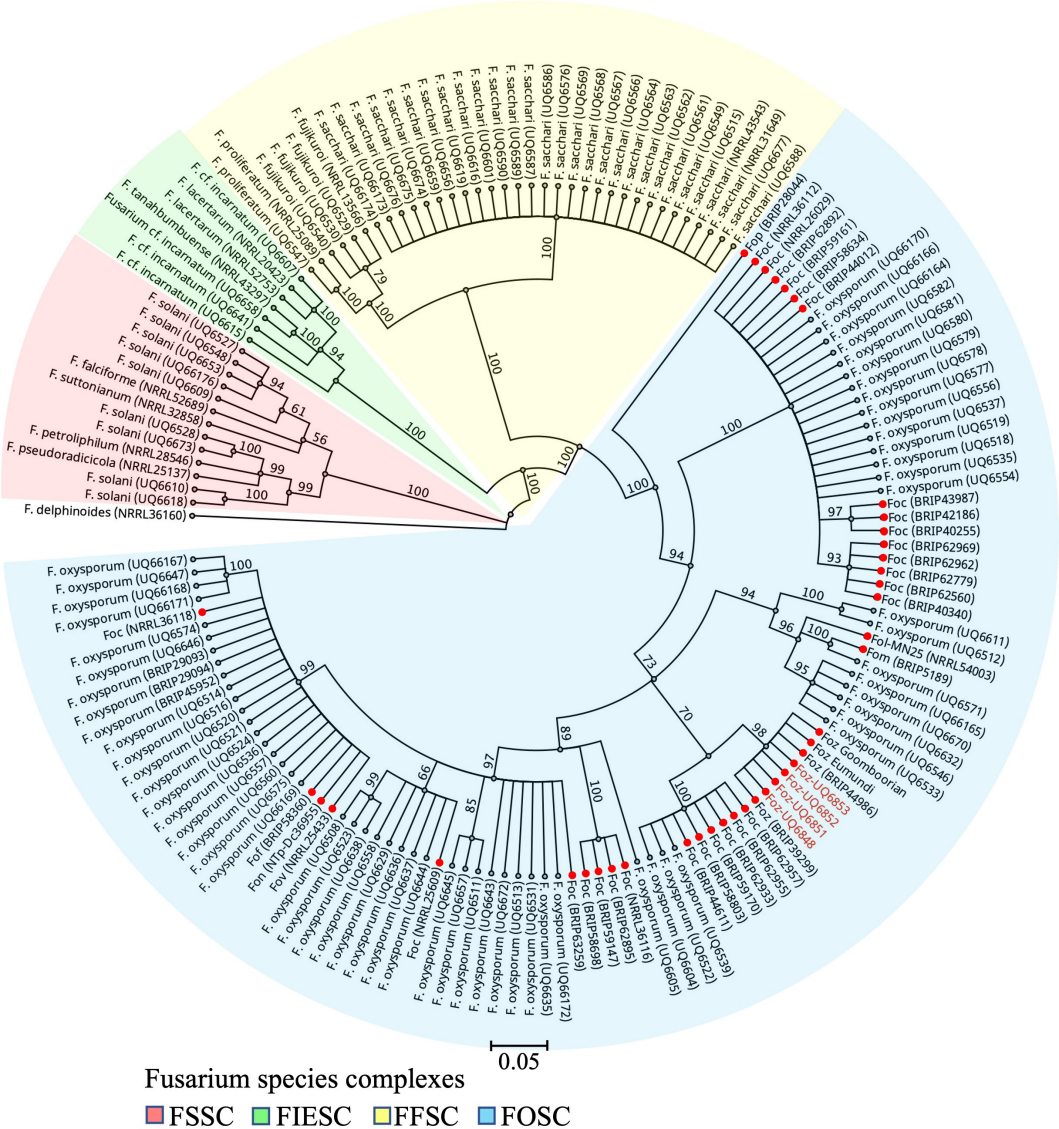
Determination of the phylogenetic positions of *Foz* isolates UQ6848, UQ6851, UQ6852 and UQ6853 within the *Fusarium oxysporum* species complex using translation elongation factor 1-alpha (TEF-1α). The four isolates are highlighted in red. The four *Fusarium* species complexes presented in this Bayesian inference phylogeny include the *Fusarium incarnatum-equiseti* species complex (FIESC); the *Fusarium fujikuroi* species complex (FFSC); the *Fusarium oxysporum* species complex (FOSC); and the *Fusarium solan*i species complex (FSSC). Where it is known, the *formae speciales* of pathogenic isolates within FOSC are indicated with a red circle. Branch labels indicate the posterior probability as a percentage. A scale range of 0.05 is indicated below the tree.

*TEF-1α* sequencing showed that UQ6848 (NCBI GenBank: OR425153), UQ6851 (OR425154), UQ6852 (OR425155) and UQ6853 (OR425156) shared 100 % nucleotide identity with each other, as well as with three other known *Foz* isolates (*Foz* Goomboorian, *Foz* Eumundi, *Foz* BRIP44986). Their sequences differ from that of *Foz* BRIP39299 by three SNPs and a single base INDEL. The phylogenetic tree reconstructed using Bayesian inference produced a topology that separated the four species complexes of *Fusarium* (Fig. 2). This phylogeny was presented, and its topology described in a previous study [18]. Within the *Fusarium oxysporum* species complex (FOSC), all four isolates used in this study are clustered together in a subclade with known *Foz* isolates, including *Foz* Goomboorian, *Foz* Eumundi, *Foz* BRIP44986 and *Foz* BRIP39299 (Fig. 2). The sequences and the phylogenetic positions of these four isolates within FOSC confirmed their *Foz* identity.

**Fig. 3.**
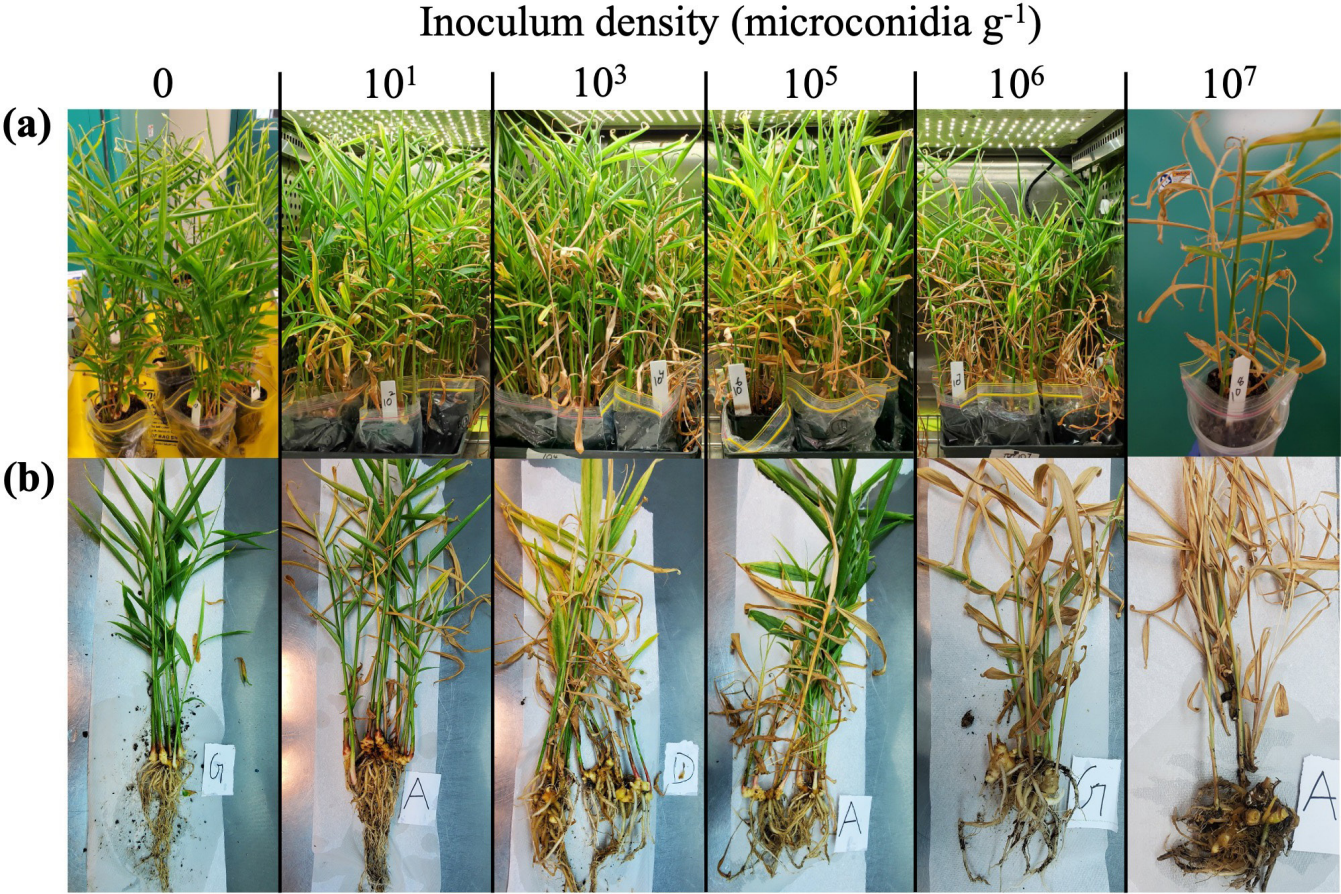
A qualitative assessment of external symptoms of Fusarium yellows on *Foz*-susceptible ginger cultivar Canton. Plants were examined at 8 weeks post-inoculation. (**a**) Potted ginger cultivar Canton plants at the time of harvest. (**b**) Individual representative plants from each treatment with above and below ground level portions cleaned and then visualized.

Inoculum concentration had a discernible effect on the severity of Fusarium yellows in ginger cultivar Canton (Fig. 3). *Foz*-inoculated plants could be easily differentiated from uninoculated controls by the development of yellowing symptoms typical of *Foz* infection (Fig. 3a). The degree of senescing leaves and tillers was positively associated with the inoculum dosage (Fig. 3b), with sporadic yellowing of stems and leaves observed at the low-to-medium inoculum concentration (10^1^, 10^3^, 10^5^ microconidia g^−1^ soil) in comparison to the uninoculated controls which showed little-to-no yellows. *Foz* susceptibility was exacerbated at the high inoculum levels (10^6^, 10^7^ microconidia g^−1^ soil), where tiller abortions due to Fusarium yellows occurred at a relatively high rate and the whole root systems appeared discoloured (Fig. 3b).

**Fig. 4.**
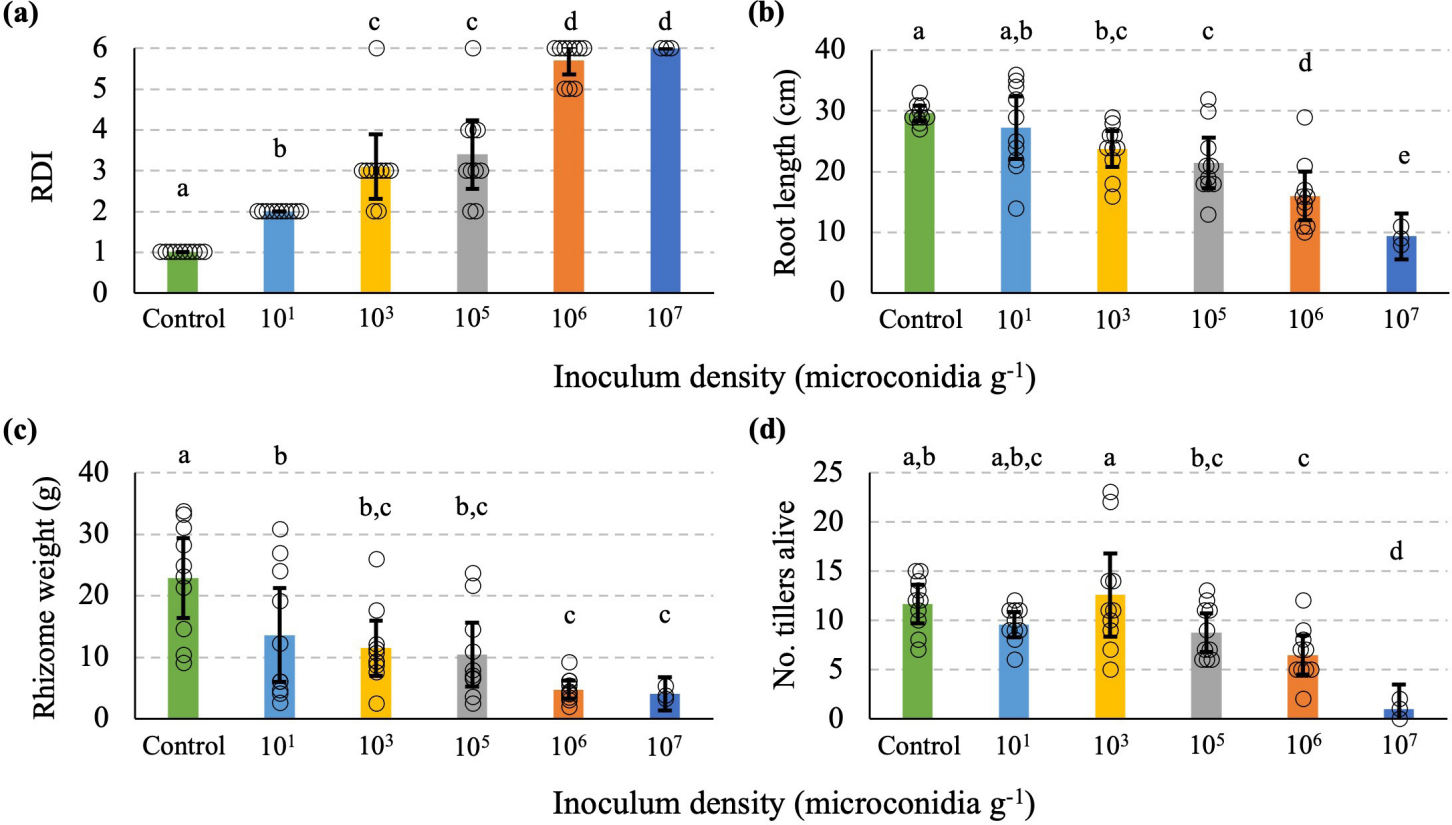
Characterization of *Foz*-infected ginger cultivar Canton plants at 8 weeks post-inoculation. (**a**) Rhizome discolouration index (RDI) scored using a 1–6 scale: 1, clean rhizome; 2, >0 and <=10 %; 3, >10 % and <=25 %; 4, >25 % and <=50 %; 5, >50 % and <=75 %; 6, >75 % and <=100 % discolouration. (**b**) Root length in centimetres of the longest adventitious root of a rhizome node. (**c**) Weight of the entire rhizome of the ginger plants. (**d**) Number of tillers that were still green or alive at the time of harvest. Control, uninoculated plants. The treatments were 10^1^, 10^3^, 10^5^, 10^6^ and 10^7^ of microconidia g^−1^ soil. *n*=10 for each treatment from 10^1^ to 10^6^, *n*=3 for 10^7^. The errors bars indicate 95 % confidence intervals of the means. Means for each treatment group were pairwisely seperated by Duncan’s multiple range test at α=0.05. Matching superscript letters above data columns (Duncan grouping) indicate that their means are not significantly different from one another at p ≤ 0.05.

Internally, rhizome discolouration was positively and quantitatively associated with the inoculum level (Fig. 4a). While the uninoculated controls were completely clean, the 10^1^ microconidia g^−1^ of soil dose produced a low but significant level discolouration with a mean score of 2 (<10 %). This difference is also associated with a significant reduction in rhizome size but not with root length or number of tillers alive (Fig. 4b-d). This suggests that a low dosage of *Foz* spores can still infect the rhizome, potentially reducing the crop yield despite plants inoculated at low spore concentrations having a similar number of tillers or length of root systems as uninoculated controls. The rhizome discolouration index (RDI) provided the best sensitivity for differentiating the dose levels amongst the four traits scored (Fig. 4a). RDI differentiated the low dose 10^1^ microconidia g^−1^ soil from that of control, as well as differentiating medium doses of 10^3^/10^5^ microconidia g^−1^ soil from the high doses 10^6^/10^7^ microconidia g^−1^ soil. This quantitative effect of discolouration is negatively associated with a reduction in root length and rhizome weight (Fig. 4b). However, rhizome weight seems to be most sensitive to *Foz* infection, with 10^3^, 10^5^, 10^6^, 10^7^ microconidia g^−1^ soil treatments all suffering similar weight reductions (Fig. 4c).

Furthermore, reisolation of *Foz* showed that it was present in the rhizome of all treatment groups and in the roots of all except the plants subjected to the spore concentration of 10^1^ microconidia g^−1^ soil (Table 1). The alive tillers count was the least affected by *Foz*, with a significant reduction only observed in 10^6^ microconidia g^−1^ soil or higher dosage (Fig. 4d). This is consistent with the presence of *Foz* detected in the tillers of treatment groups 10^6^ and 10^7^ microconidia g^−1^ soil but not in the other treatments or the control (Table 1). These observations demonstrate that the external symptoms above ground may not necessarily reflect the reduction in rhizomes and roots below ground at low *Foz* doses. At the high doses of 10^6^ and 10^7^ microconidia g^−1^ soil, the saturation of spores in the soil allowed plants to be uniformly infected, leading to the wilting of whole plants or even plant death. Evaluation methods for screening *Fusarium* wilt resistance have been available for other plant species such as cotton and lettuce [3, 23], but the absence of a *Fusarium* screening method for ginger has hampered disease research. A previous study also demonstrated that the spore concentrations of fungal pathogens have an effect on the wilt symptoms of ginger [8]. In comparison, the data generated in this study show that ginger can respond to *Foz* at a lower or higher dose than 10^3^–10^5^ microconidia g^−1^ soil, allowing sensitivity in host responses against *Foz* to be refined.

**Table 1.** Three plants randomly selected from each treatment group (*n*=10, labelled A to J for each treatment from 10^1^ to 10^6^, *n*=3 for 10^7^) were used to reisolate *Foz* from the rhizome, roots and tillers. ‘+’ indicates a positive colony that conformed to a typical *F. oxysporum* f. sp. *zingiberi* morphology [9, 32] and ‘−‘ indicates the absence of any *F. oxysporum*-like colonies

Treatment	Plant	Rhizome	Roots	Tillers
Control	B	−	−	−
Control	D	−	−	−
Control	F	−	−	−
10	C	+	−	−
10	E	+	−	−
10	H	+	−	−
10^3^	F	+	+	−
10^3^	I	+	−	−
10^3^	J	+	−	−
10^5^	A	+	+	−
10^5^	E	+	+	−
10^5^	F	+	−	−
10^6^	A	+	−	+
10^6^	B	+	+	−
10^6^	G	+	+	−
10^7^	A	+	+	+
10^7^	B	+	−	+
10^7^	C	+	+	−

Management of soil-borne pathogens is a significant challenge for ginger production in Australia, with diseases such as Fusarium yellows, Pythium rot caused by *Pythium myriotylum* [6] and root-knot nematodes (*Meloidogyne incognita* and *M. javanica*) [24, 25] requiring a combination of management techniques, including fallowing land, improving soil health, managing drainage and the use of biological control agents and clean rhizomes for planting [26]. The application of biocontrol control agents such as *Trichoderma* has the potential to control and reduce *Fusarium* spp. and other soil phytopathogens [27]. One of the key aspects of controlling Fusarium yellows is the improvements in minimizing the inoculum pressure for diseases in the field by using clean planting materials and schemes through tissue culture [28] or using material produced from clean mother blocks. In Australia, ginger is produced mainly on red ferrosol soils and is managed intensively with inputs and tillage during multiple annual production cycles before the field is rotated to fallow crops [3, 29, 30]. When disease outbreaks occur, it is important to understand the infection process and disease threshold so that *Foz* can be diagnosed and monitored through immunological, phylogenetic and pathogenicity-related approaches [31].

This study has developed a rigorous methodology to test the virulence of *Foz* isolates on ginger plants by establishing a minimum threshold level of microconidia per gram of soil required for infection in a controlled environment. This screening method can be used to assess *Foz* resistance levels in wild relatives and cultivars of ginger. Rhizome discolouration offers a reliable trait marker for screening ginger’s susceptibility to *Foz*. Therefore, it can be used to rapidly fast track elite resistance phenotypes for introduction into commercial ginger cultivars. Further optimizations can potentially allow this method to be adapted to screen for other plant–soil phytopathogen interactions.

Plant breeding for disease resistance is a part of plant disease management. Integrated management practices, including improvements in soil health, addition of organic amendments such as poultry manure and sawdust, annual rotations of suitable cover crops and prolonged fallow between ginger cultivations will help reduce but not completely eliminate *Foz* in the soil [29, 30]. Even with management practices applied, re-emergence of the disease can potentially override biological suppression developed through improved soil health [3]. It is therefore important to ensure, where possible, that cropping soil used for ginger is not contaminated with soil-borne pathogens such as *Foz*, as it has been demonstrated that even low numbers of microconidia per gram of soil can still result in significant loss of rhizome quality and quantity, and therefore crop yield.
